# Lipoprotein(a) concentration, kringle IV-2 repeat copy number, and myocardial infarction risk in Chinese populations: insights from the INTERHEART China study

**DOI:** 10.1186/s12944-026-02929-y

**Published:** 2026-04-25

**Authors:** Jinsong Jiang, Xiaonan Ma, Ying Wei, Binong Su, Xin Liu, Yanyan Xiao, Yan Gu, Ziyu Wang, Dong Luo, Huafang Gao, Xingyu Wang, Aihua Hu

**Affiliations:** 1 Department of Non-Communicable Disease Management, Beijing Children’s Hospital, Capital Medical University, National Center for Children’s Health, Beijing, China; 2 National Human Genetic Resources Center, National Research Institute for Family Planning, Beijing, China; 3 Chinese Academy of Medical Sciences & Peking Union Medical College, Beijing, China; 4 Beijing Hypertension League Institute, Beijing, China; 5 Department of Cardiology, Beijing Children’s Hospital, Capital Medical University, National Center for Children’s Health, Beijing, China

**Keywords:** Lipoprotein(a), Kringle IV-2 repeat copy number, Myocardial infarction, Chinese population, Quantitative PCR (qPCR)

## Abstract

**Objective:**

To elucidate the independent and joint effects of plasma Lipoprotein(a) [Lp(a)] concentration and Kringle IV-2 (KIV-2) repeat copy number in genomic DNA on the risk of acute myocardial infarction (AMI) in a Chinese population, to estimate their population attributable risks (PAR), and to explore interactions with metabolic and psychosocial factors, thereby providing evidence for precise cardiovascular prevention and control.

**Methods:**

Based on the INTERHEART China subgroup case-control study, 4,479 participants from 26 centers in China were included (2,100 first-onset AMI patients, 2,379 age- and sex-matched controls). Risk factors (lifestyle, metabolic indicators, psychosocial factors) were collected using standardized questionnaires. Lp(a) concentration was measured using an isoform-insensitive immunoassay, and KIV-2 repeat copy number in genomic DNA was quantified using quantitative PCR (qPCR) (represented as ΔCT value, lower values indicate fewer repeats). Multivariable logistic regression models were used to analyze risk associations, restricted cubic splines assessed dose-response relationships, PAR was calculated, and interactions were tested.

**Results:**

Elevated Lp(a) concentration (highest vs. lowest quintile; OR = 1.784, 95% CI: 1.402–2.270) and fewer KIV-2 repeats (lowest vs. highest quintile; OR = 2.421, 95% CI: 1.905–3.086) were significantly associated with AMI risk. Dose-response analysis revealed that Lp(a) concentration showed a monotonically increasing relationship with AMI risk, while KIV-2 repeats showed a continuous negative association. Both factors remained independent predictors after mutual adjustment, and were moderately negatively correlated (*r*=-0.323, *P* < 0.001). The PAR was 10.3% for the highest Lp(a) quintile (> 17.6 mg/dL) and 10.4% for the lowest KIV-2 repeat quintile (ΔCT < 4.1). No significant interactions were found between Lp(a) or KIV-2 and sex, diabetes, smoking, or psychological factors (P-interaction > 0.00625).

**Conclusion:**

In the Chinese population, both Lp(a) concentration and KIV-2 repeat copy number in genomic DNA independently predict AMI risk, with PARs exceeding 10% for both. This study reveals for the first time that KIV-2 repeat copy number may have pathophysiological implications independent of Lp(a) concentration regulation, suggesting the need to incorporate this genetic marker into high-risk screening and to adopt a lower Lp(a) risk threshold of > 18 mg/dL (approximately the 80th percentile) for this population.

**Supplementary Information:**

The online version contains supplementary material available at 10.1186/s12944-026-02929-y.

## Introduction

Cardiovascular disease (CVD) is the leading cause of global mortality and loss of disability-adjusted life years, with its burden continuously rising in low- and middle-income countries (LMICs) [1]. China, as the world’s most populous country, is experiencing a rapid epidemic of coronary heart disease (CHD), but its risk factor profile shows significant regional and ethnic specificity: for instance, average serum total cholesterol levels in the Chinese population are generally lower than in Western populations [2], while diabetes and psychosocial factors exert stronger risk effects for myocardial infarction (MI) [3, 4].

Lipoprotein(a) [Lp(a)] is an atherogenic particle synthesized by the liver, its plasma concentration is primarily determined by the copy number variation (CNV) of the kringle IV type 2 (KIV-2) domain in the LPA gene [5, 6]. Fewer KIV-2 repeats encode smaller apolipoprotein(a) [apo(a)] isoforms, which are associated with higher Lp(a) concentrations and cardiovascular risk [7]. Notably, the median Lp(a) concentration in the Chinese population (7.8 mg/dL) is significantly lower than in African (27.2 mg/dL) and European populations (11.5 mg/dL), and apo(a) isoforms are larger (median 28 vs. 24 in Africa) [8], which may influence the risk predictive value of Lp(a) in this population.

Moreover, many studies have focused on the association between the size of apo(a) protein isoforms estimated by immunoblotting and the risk of MI [9]. However, the KIV-2 repeat copy number in genomic DNA and its final protein expression are not entirely consistent due to the regulation of complex processes such as transcription, translation, and secretion efficiency. Therefore, whether direct detection of KIV-2 repeats in genomic DNA can provide risk information beyond that of protein isoform size remains unclear, particularly in the Chinese population. Although quantitative PCR (qPCR) offers a high-throughput method for KIV-2 repeats and has been correlated with immunoblotting results [10], its application for risk prediction has not been systematically evaluated in this specific population.

Based on the standardized framework of the global INTERHEART study [11], this study integrates phenotypic and genotypic data from 4,479 Chinese participants (2,100 AMI patients, 2,379 controls), aiming to: elucidate the independent and joint effects of Lp(a) concentration and qPCR-quantified KIV-2 repeat copy number in genomic DNA on MI risk in the Chinese population; estimate the population attributable risk (PAR) of Lp(a)-related indicators and traditional risk factors; explore interactions with metabolic and psychosocial factors. The findings will provide critical evidence for precise cardiovascular prevention strategies in China.

## Methods

### Study design and population

**Fig. 1:**
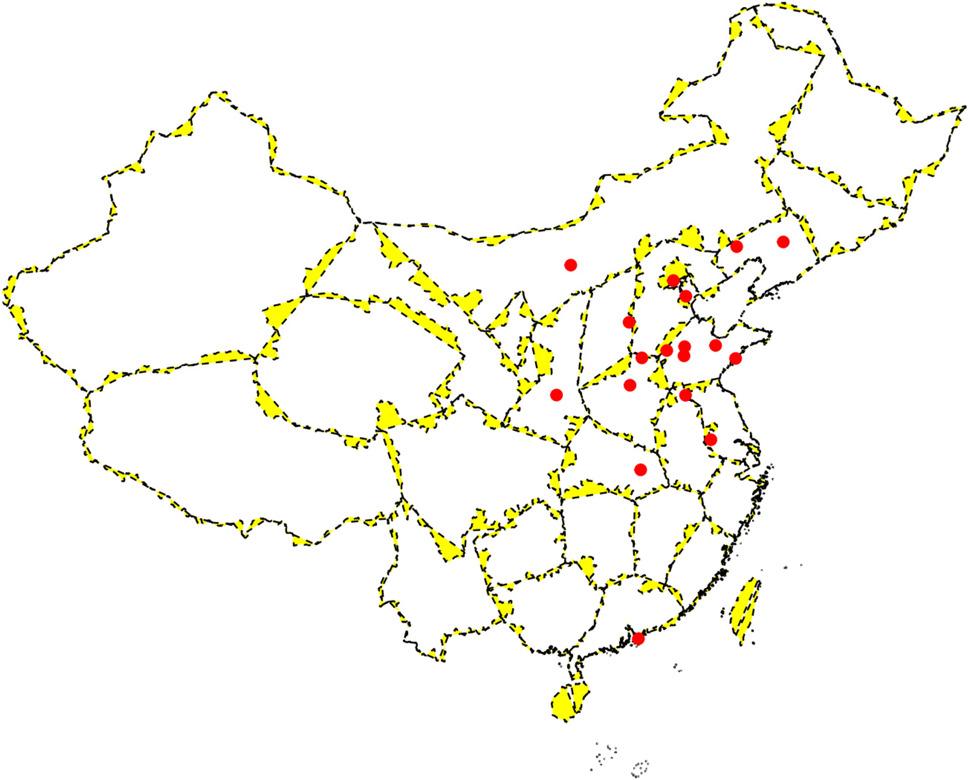
Recruiting centres from the INTEHEART study by region in China

This study is based on data from the China subgroup of the global INTERHEART study [4]. First-onset acute myocardial infarction (AMI) patients and age (± 5 years), sex-matched controls were enrolled from 26 centers in China (19 northern, 7 southern; Fig. 1). Case group (*n* = 2100): met WHO AMI diagnostic criteria (typical symptoms + new ECG ischemic changes/elevated cardiac enzymes), hospitalized within 24 h of symptom onset. Control group (*n* = 2379): no history of CHD, recruited from non-cardiac inpatients or their family members at the same centers [1]. Exclusion criteria: previous myocardial infarction, active malignancy, end-stage renal disease. This study complied fully with the Declaration of Helsinki, Good Clinical Practice guidelines, and relevant local laws and regulations. The study was approved by the ethics committees of all participating centers (Approval No.: EC-2004-CHN-001), and all participants provided written informed consent.

### Data collection and standardized procedures

Globally unified structured questionnaires and physical examination protocols were used [4]. *Questionnaire survey*: Covered demographic characteristics, socioeconomic status (education, household income), lifestyle (smoking, alcohol consumption, physical activity), dietary patterns (categorized per Supplementary Table 1), cardiovascular history, and psychosocial factors (depression, stressful events). Physical measurements: Height, weight, waist circumference (narrowest point between the costal margin and iliac crest), hip circumference (widest point around the hips), body mass index (BMI) and waist-to-hip ratio (WHR) were calculated.

*Key variable definitions*: Current smoking: Smoked within the past 12 months (including those who quit within the last year). Regular exercise: ≥4 h of moderate/high-intensity activity per week. Regular alcohol consumption: Drinking alcohol ≥ 3 times per week. Hypertension or diabetes: Based on self-reported history (as acute-phase blood pressure/glucose in cases was affected by treatment) [4]. Specific life events were defined as experiencing any of the following in the past year: marital separation or divorce, unemployment or retirement, crop loss or business failure, violence, major intra-family conflict, major personal injury or illness, death or major illness of a close relative, death of a spouse, or other major stress. Depression was assessed by asking participants if they felt sad, blue, or depressed for two weeks or longer consecutively in the past 12 months. Stress was defined as feeling irritable, filled with anxiety, or having difficulty sleeping due to work or home conditions [12]. For waist-to-hip ratio, tertiles were calculated based on the overall control data separately for males and females. For ApoB/ApoA1 ratio, LDL-C/HDL-C ratio, Lp(a), and number of KIV2 repeats by qPCR, quintiles were calculated based on the overall control data.

### Biological sample testing

*Blood collection and storage*: Non-fasting blood samples were centrifuged and aliquoted within 2 h of collection, stored at -70 °C (Chinese centers), and periodically transported in liquid nitrogen to the Beijing laboratory [2, 4].

*Biochemical indicator testing*: (1) Lipid profile: Total cholesterol, HDL-C (enzymatic method); LDL-C (Friedewald formula); Apolipoprotein A1 (ApoA1), B (ApoB) (immunoturbidimetry, Roche/Hitachi 911 analyzer) [4]. (2) Lp(a) concentration: Measured using an isoform-insensitive immunoassay (Denka Seiken kit), avoiding interference from KIV-2 repeat number on measured values [8]. (3) HbA1c: High-performance liquid chromatography.

*KIV-2 repeat number genotyping*: (1) DNA extraction: Genomic DNA from white blood cells (QIAamp DNA Blood Kit). (2) qPCR assay: Following the protocol by Lanktree et al. [10], using TaqMan probes targeting exons 4/5 of the LPA gene KIV-2 domain (sequences in Supplementary Table 2), with RNase P as a single-copy internal reference. (3) Repeat number calculation: Total KIV-2 repeat number was estimated based on ΔCT (target gene CT – reference CT). Specific formula: ΔCT was calculated for the exon 4 (ΔCT 4) and exon 5 (ΔCT 5) probes for all individuals, and the difference between ΔCT 4 and ΔCT 5 (ΔΔCT) was calculated; individuals with ΔΔCT greater than two standard deviations from the mean were excluded from analysis. The average of ΔCT 4 and ΔCT 5 was then used for further analysis as the relative Kringle repeat number [10].

### Statistical analysis

Categorical data were analyzed using chi-square tests. Continuous variables were summarized using means and medians and compared using t-tests or appropriate non-parametric tests. When data were categorized into tertiles or quintiles, these categories were based on the overall control data. For waist-to-hip ratio, sex-specific cutoffs were used.

*Primary analysis methods*: (1) Association testing: Unconditional logistic regression calculated OR (95% CI), with stepwise adjustment for confounders: Model 1: Unadjusted; Model 2: Adjusted for age, sex; Model 3: Additionally adjusted for all other risk factors. (2) Dose-response relationship: Restricted cubic spline models (R packages mgcv, ggplot2, dplyr) analyzed the nonlinear association between Lp(a) concentration, KIV-2 repeats, and AMI risk, with confidence intervals calculated by the Delta method. Age, sex, and ApoB/ApoA1 ratio were included as covariates, adjusting for risk factors. (3) Interaction: Stratified analysis tested subgroup differences (sex, diabetes, etc.), with Bonferroni-corrected significance threshold (P-interaction < 0.00625). For subgroup analysis of the association between Lp(a) levels/qPCR-quantified KIV-2 repeats and MI, adjustments were made for age, sex, and ApoB/ApoA1 ratio. (4) Population attributable risk (PAR): PAR and 95% CI were calculated using functions (R packages foreign, dplyr, boot), with the Delta method and logit transformation for more stable confidence intervals. For protective factors (daily fresh vegetables and fruits, exercise), PAR for the non-exposed group was calculated.

All analyses were performed using R 4.5.0 and SAS 9.4. The 2-tailed *P* < 0.05 was defined statistically significant.

## Results

### Population baseline characteristics

A total of 4,479 Chinese participants were included (2,100 AMI patients, 2,379 controls). Baseline characteristics are shown in Table 1. Demographics and metabolic indicators: The mean age of AMI patients was 61.08 ± 11.77. The prevalence of diabetes (12.1% vs. 3.2%) and hypertension (38.5% vs. 22.9%) was significantly higher in the case group (both *P* < 0.001). Regarding lipid profile differences, the case group had significantly higher ApoB/ApoA1 ratio (0.70 ± 0.22 vs. 0.61 ± 0.20), LDL-C/HDL-C ratio (2.70 ± 1.32 vs. 2.30 ± 1.18), and significantly lower HDL-C (1.05 ± 0.32 vs. 1.11 ± 0.36 mmol/L) (all *P* < 0.001). Lp(a) and genetic characteristics: The case group had higher Lp(a) concentration (16.32 ± 19.85 vs. 12.50 ± 14.76 mg/dL, *P* < 0.001) and lower qPCR-quantified KIV-2 repeat number (4.41 ± 0.42 vs. 4.52 ± 0.42, *P* < 0.001).

**Table 1:** Baseline Characteristics of Chinese Participants in the INTERHEART Study

				Total (n=4479)	Controls (n=2379)	Cases (n=2100)	P value
General characteristics							
Age (years)				60.44±11.58	59.87±11.37	61.08±11.77	<0.001
Sex (male)				3120 (69.7)	1644 (69.1)	1476 (70.3)	0.391
BMI (kg/m^2^)				24.52±3.04	24.39±2.99	24.67±3.09	0.002
Waist to hip ratio				0.87±0.08	0.87±0.08	0.88±0.08	0.288
Systolic BP (mmHg)				125.11±18.85	127.84±17.54	121.99±19.80	<0.001
Diastolic BP (mmHg)				77.98±10.91	79.36±9.80	76.39±11.87	<0.001
Metabolic factors							
ApoA1 (G/L)				1.35±0.30	1.41±0.32	1.29±0.27	<0.001
ApoB (G/L)				0.85±0.25	0.83±0.23	0.88±0.26	<0.001
ApoB/ApoA1 ratio				0.65±0.22	0.61±0.20	0.70±0.22	<0.001
HbA1c (%)				5.98±1.07	5.89±0.91	6.09±1.22	<0.001
Total cholesterol (mmol/L)				4.62±1.17	4.57±1.16	4.67±1.18	0.009
TG (mmol/L)				1.64±1.07	1.68±1.07	1.60±1.07	0.023
LDL-C (mmol/L)				2.47±0.89	2.35±0.85	2.60±0.91	<0.001
HDL-C (mmol/L)				1.08±0.34	1.11±0.36	1.05±0.32	<0.001
TG/LDL-C ratio				0.73±0.54	0.79±0.57	0.67±0.51	<0.001
LDL-C/HDL-C ratio				2.49±1.26	2.30±1.18	2.70±1.32	<0.001
Lp(a) mg/dl				14.29±17.44	12.50±14.76	16.32±19.85	<0.001
number of KIV2 repeats by qPCR				4.47±0.42	4.52±0.42	4.41±0.42	<0.001
History of disease							
Diabetes				330 (7.4)	75 (3.2)	255 (12.1)	<0.001
High blood pressure				1352 (30.2)	544 (22.9)	808 (38.5)	<0.001
Psychosocial factors							
Stress at work or home, Permanent				96 (2.1)	33 (1.4)	63 (3.0)	<0.001
Financial stress, Severe				552 (12.3)	292 (12.3)	260 (12.4)	0.914
Specified life events (Two or more events)				196 (4.4)	70 (2.9)	126 (6.0)	<0.001
Depression				576 (12.9)	208 (8.7)	368 (17.5)	<0.001
Socioeconomic status							
Education							
Fewer than 8 years				2223 (49.6)	1070 (45.0)	1153 (54.9)	<0.001
College or university				993 (22.2)	599 (25.2)	394 (18.8)	<0.001
Financial							
Annual household income							
< 4,000 yuan per year				520 (11.6)	261 (11.0)	259 (12.3)	0.156
≥ 20,000 yuan per year				466 (10.4)	266 (11.2)	200 (9.5)	0.070
Both house and car				163 (3.6)	79 (3.3)	84 (4.0)	0.226
Occupation							
Workers in general, farmers				2067 (46.1)	1106 (46.5)	961 (45.8)	0.626
Technician, police/military, secretary				1241 (27.7)	650 (27.3)	591 (28.1)	0.540
Professionals, businessmen, private owners				756 (16.9)	416 (17.5)	340 (16.2)	0.248
Lifestyle factors							
Current smoking				1552 (34.7)	656 (27.6)	896 (42.7)	<0.001
Alcohol				712 (15.9)	368 (15.5)	344 (16.4)	0.405
Exercise				576 (12.9)	350 (14.7)	226 (10.8)	<0.001
Dietary patterns							
Fresh vegetables				2984 (66.6)	1626 (68.3)	1358 (64.7)	0.009
Pickled vegetables (brine)				978 (21.8)	545 (22.9)	433 (20.6)	0.064
Fresh fruit or juice				1957 (43.7)	1157 (48.6)	800 (38.1)	<0.001
Whole grains				1756 (39.2)	942 (39.6)	814 (38.8)	0.568
Fish and other seafood				2740 (61.2)	1469 (61.7)	1271 (60.5)	0.401
Deep fried food/snacks/fast food				1717 (38.3)	885 (37.2)	832 (39.6)	0.097
Desserts/sweet snacks				439 (9.8)	212 (8.9)	227 (10.8)	0.033
Bean and bean products				599 (13.4)	323 (13.6)	276 (13.1)	0.670

Categorical variables were summarized as frequencies and percentages, while continuous variables were expressed as mean ± standard deviation or median (interquartile range)

*BMI* body mass index, *ApoA1* Apolipoprotein A-1, *ApoB* Apolipoprotein B, *HbA1c* glycated hemoglobin, *TG* triglycerides, *LDL-C* low-density lipoprotein cholesterol, *HDL-C* high-density lipoprotein cholesterol, *Lp(a) *Lipoprotein(a)

### Strength of association between risk factors and AMI

Odds ratios for risk factors after multivariable adjustment are shown in Table 2. Strongest risk factors: Diabetes (OR = 3.733, 95% CI: 2.68–5.185), depression (OR = 2.525, 95% CI: 2.019–3.157), permanent work or home stress (OR = 2.512, 95% CI: 1.320–4.777), and current smoking (OR = 2.333, 95% CI: 1.968–2.767). Lp(a) and genetic indicators: Lp(a) concentration (highest vs. lowest quintile): OR = 1.784 (95% CI: 1.402–2.270). KIV-2 repeat number (lowest vs. highest quintile): OR = 2.421 (95% CI: 1.905–3.086), indicating fewer repeats confer higher risk. Protective factor: Daily intake of fresh vegetables and fruits significantly reduced risk (OR = 0.596, 95% CI: 0.510–0.697).

**Table 2:** Risk of acute myocardial infarction associated with individual risk factors in China

Risk factor		Controls (%)	Cases (%)	Model 1		Model 2		Model 3	
				Odds ratio (95% CI)	*P* value	Odds ratio (95% CI)	*P* value	Odds ratio (95% CI)	*P* value
Current smoking		27.6	42.7	1.958 (1.729 ~ 2.219)	< 0.001	2.399 (2.084 ~ 2.762)	< 0.001	2.333 (1.968 ~ 2.767)	< 0.001
Diabetes		3.2	12.1	4.248 (3.260 ~ 5.535)	< 0.001	4.248 (3.257 ~ 5.541)	< 0.001	3.733 (2.687 ~ 5.185)	< 0.001
Hypertension		22.9	38.5	2.111 (1.854 ~ 2.404)	< 0.001	2.082 (1.826 ~ 2.374)	< 0.001	2.009 (1.706 ~ 2.366)	< 0.001
Waist to hip ratio									
(2 vs. 1)		33.3	34.4	1.114 (0.964 ~ 1.288)	0.143	1.117 (0.966 ~ 1.291)	0.137	1.225 (1.029 ~ 1.459)	0.022
(3 vs. 1)		33.3	34.8	1.127 (0.975 ~ 1.302)	0.106	1.122 (0.970 ~ 1.298)	0.122	1.134 (0.947 ~ 1.358)	0.172
Fresh vegetables and fruit daily		38.7	28.9	0.645 (0.569 ~ 0.731)	< 0.001	0.639 (0.563 ~ 0.725)	< 0.001	0.596 (0.510 ~ 0.697)	< 0.001
Exercise		14.7	10.8	0.696 (0.582 ~ 0.832)	< 0.001	0.679 (0.567 ~ 0.813)	< 0.001	0.680 (0.545 ~ 0.848)	0.001
Alcohol intake		15.5	16.4	1.077 (0.918 ~ 1.265)	0.362	1.127 (0.952 ~ 1.335)	0.164	1.037 (0.840 ~ 1.281)	0.735
ApoB/ApoA1 ratio									
(2 vs. 1)		20.0	12.9	1.192 (0.943 ~ 1.507)	0.142	1.181 (0.934 ~ 1.493)	0.165	0.975 (0.742 ~ 1.282)	0.857
(3 vs. 1)		20.0	16.1	1.485 (1.183 ~ 1.864)	0.001	1.479 (1.177 ~ 1.857)	0.001	1.126 (0.844 ~ 1.502)	0.421
(4 vs. 1)		20.0	22.9	2.116 (1.700 ~ 2.633)	< 0.001	2.082 (1.672 ~ 2.592)	< 0.001	1.596 (1.182 ~ 2.156)	0.002
(5 vs. 1)		20.0	37.4	3.455 (2.799 ~ 4.264)	< 0.001	3.423 (2.772 ~ 4.227)	< 0.001	2.232 (1.620 ~ 3.076)	< 0.001
Depression		8.7	17.5	2.218 (1.850 ~ 2.659)	< 0.001	2.229 (1.858 ~ 2.673)	< 0.001	2.525 (2.019 ~ 3.157)	< 0.001
Stress at work or home, Permanent		1.4	3.0	2.199 (1.437 ~ 3.364)	< 0.001	2.352 (1.534 ~ 3.605)	< 0.001	2.512 (1.320 ~ 4.777)	0.005
Specified life events (Two or more events)		2.9	6.0	2.105 (1.562 ~ 2.837)	< 0.001	2.091 (1.551 ~ 2.820)	< 0.001	1.744 (1.197 ~ 2.540)	0.004
Education									
Fewer than 8 years		45.0	54.9	1.491 (1.325 ~ 1.678)	< 0.001	1.510 (1.331 ~ 1.714)	< 0.001	1.516 (1.271 ~ 1.810)	< 0.001
College or university		25.2	18.8	0.687 (0.595 ~ 0.793)	< 0.001	0.690 (0.596 ~ 0.798)	< 0.001	1.004 (0.814 ~ 1.238)	0.971
LDL-C/HDL-C ratio									
(2 vs. 1)		20.0	14.6	1.285 (1.023 ~ 1.615)	0.031	1.277 (1.015 ~ 1.605)	0.037	1.000 (0.765 ~ 1.307)	0.999
(3 vs. 1)		20.0	18.3	1.602 (1.283 ~ 2.000)	< 0.001	1.592 (1.274 ~ 1.988)	< 0.001	1.165 (0.876 ~ 1.549)	0.293
(4 vs. 1)		20.0	22.9	2.009 (1.618 ~ 2.495)	< 0.001	2.020 (1.625 ~ 2.511)	< 0.001	1.187 (0.878 ~ 1.605)	0.265
(5 vs. 1)		20.0	32.8	2.889 (2.342 ~ 3.565)	< 0.001	2.947 (2.386 ~ 3.641)	< 0.001	1.300 (0.941 ~ 1.796)	0.112
Lp(a)									
(2 vs. 1)		20.1	19.0	1.556 (1.272 ~ 1.904)	< 0.001	1.545 (1.262 ~ 1.890)	< 0.001	1.534 (1.207 ~ 1.951)	< 0.001
(3 vs. 1)		20.3	19.0	1.534 (1.254 ~ 1.876)	< 0.001	1.507 (1.232 ~ 1.845)	< 0.001	1.395 (1.092 ~ 1.781)	0.008
(4 vs. 1)		19.6	21.4	1.788 (1.465 ~ 2.183)	< 0.001	1.773 (1.452 ~ 2.167)	< 0.001	1.685 (1.323 ~ 2.147)	< 0.001
(5 vs. 1)		20.0	28.3	2.321 (1.912 ~ 2.817)	< 0.001	2.299 (1.893 ~ 2.793)	< 0.001	1.784 (1.402 ~ 2.270)	< 0.001
number of KIV2 repeats by qPCR									
(2 vs. 1)		20.0	20.2	0.715 (0.598 ~ 0.854)	< 0.001	0.708 (0.592 ~ 0.847)	< 0.001	0.720 (0.582 ~ 0.890)	0.002
(3 vs. 1)		20.0	19.7	0.697 (0.583 ~ 0.833)	< 0.001	0.697 (0.583 ~ 0.834)	< 0.001	0.707 (0.570 ~ 0.877)	0.002
(4 vs. 1)		20.0	18.9	0.669 (0.559 ~ 0.801)	< 0.001	0.662 (0.553 ~ 0.793)	< 0.001	0.616 (0.495 ~ 0.766)	< 0.001
(5 vs. 1)		20.0	13.0	0.459 (0.379 ~ 0.556)	< 0.001	0.458 (0.378 ~ 0.555)	< 0.001	0.413 (0.324 ~ 0.525)	< 0.001

Model 1: unadjusted

Model 2: adjusted for age, sex

Model 3: further adjusted additionally for all other risk factor. Waist to hip ratio is based on a comparison of second or third tertiles vs. lowest tertile

ApoB/ApoA1 ratio, LDL-C/HDL-C ratio, Lp(a) and number of KIV2 repeats by qPCR are based on a comparison of second, third, fourth, or fifth quintiles vs. lowest quintile

*ApoA1* Apolipoprotein A-1, *ApoB* Apolipoprotein B, *LDL-C* low-density lipoprotein cholesterol, *HDL-C* high-density lipoprotein cholesterol, *Lp(a) *Lipoprotein(a)

### Dose-response relationship of Lp(a) concentration, KIV-2 Repeats with AMI

**Fig. 2:**
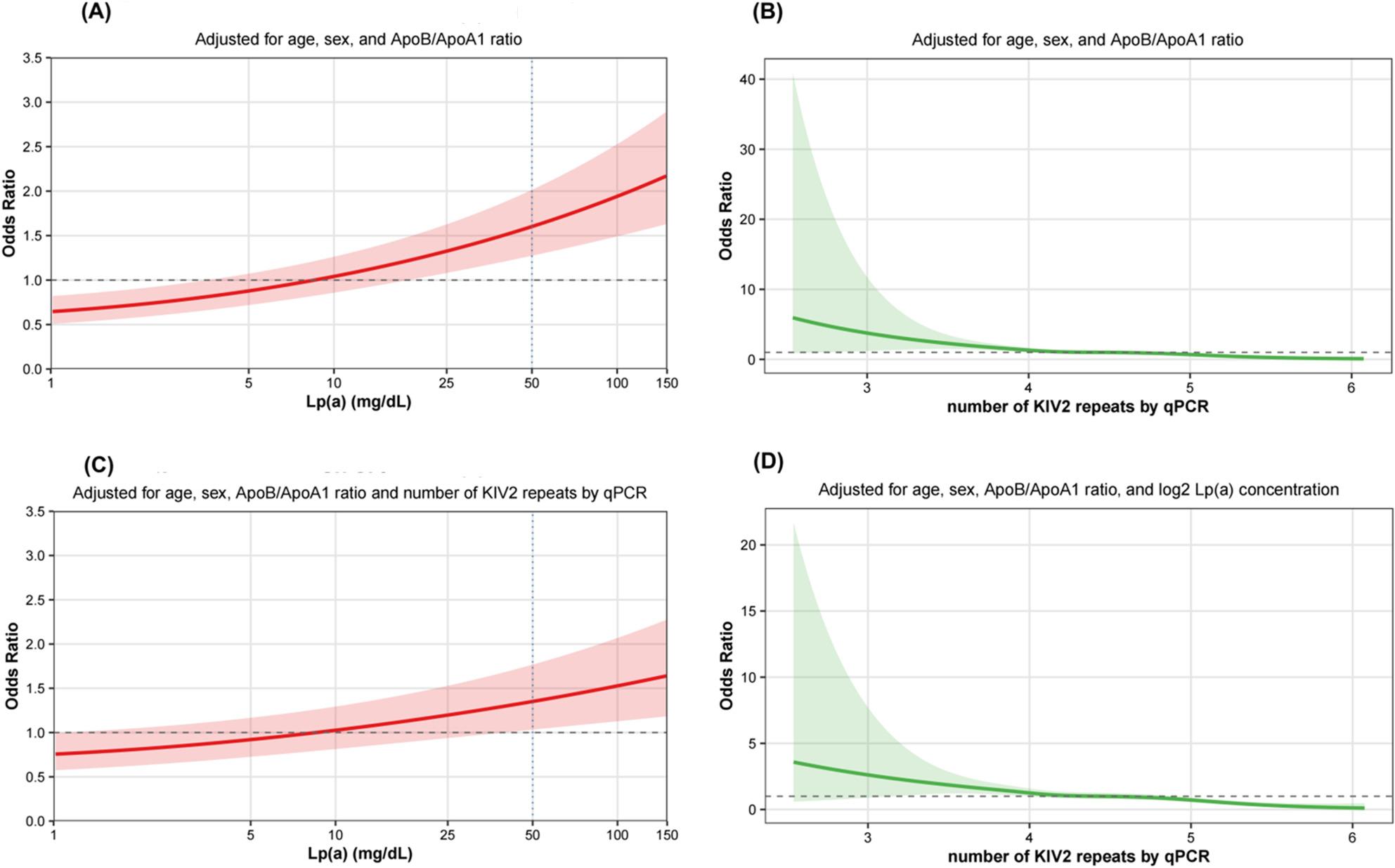
Spline analysis of myocardial infarction risk as a function of Lp(a) concentration and number of KIV2 repeats by qPCR. **A **Risk of MI as function of log2 Lp(a) concentration. **B** Risk of MI as function of number of KIV2 repeats by qPCR. **C** Risk of MI as function of log2 Lp(a) concentration. **D** Risk of MI as function of number of KIV2 repeats by qPCR

Restricted cubic spline analysis showed (Fig. 2): Lp(a) concentration had a monotonically increasing relationship with AMI risk, while KIV-2 repeats showed a negative association with AMI risk. After mutual adjustment, the associations of both Lp(a) concentration and KIV-2 repeats with AMI risk were attenuated but remained independent predictors of risk.

### Subgroup analysis and interaction

**Fig. 3:**
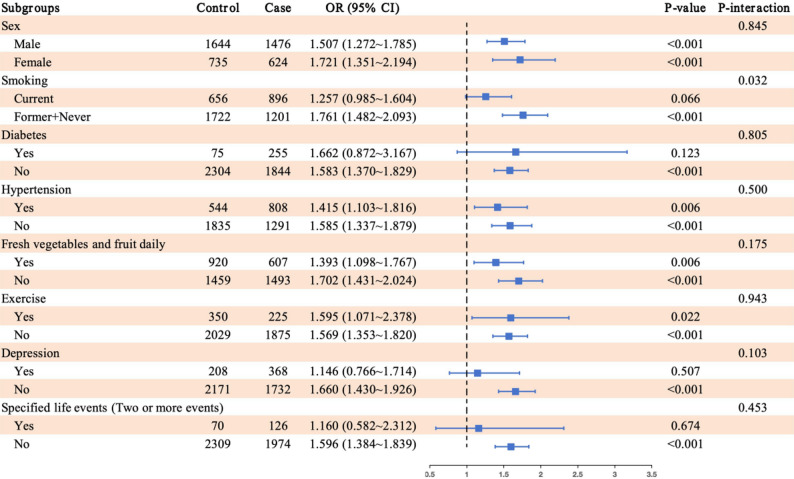
Subgroup and interaction analyses between Lp(a) and myocardial infarction across various subgroups. Note: Lp(a) levels were compared between the highest quintile vs the bottom four quintiles. Adjusted variables included age, sex, ApoB/ApoA1 ratio. ApoB/ApoA1 ratio were treated as dichotomous variables using medians. Bonferroni-adjusted α = 0.00625 (0.05/8 tests); significance: P-interaction < 0.00625

**Fig. 4:**
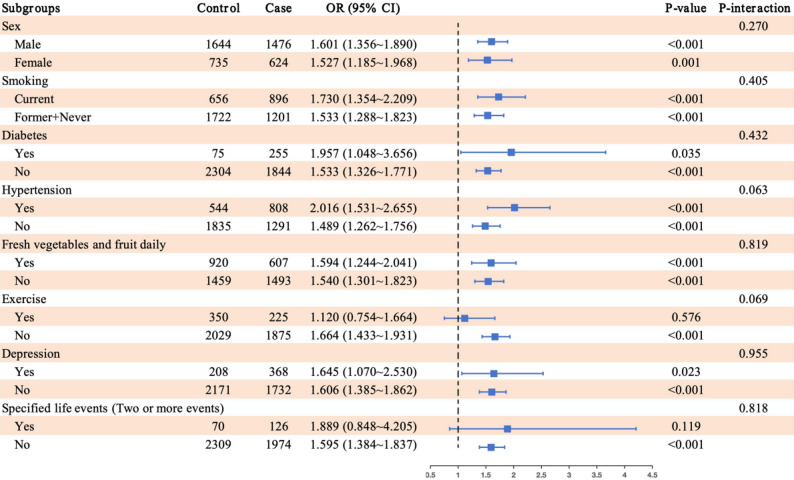
Subgroup and interaction analyses between number of KIV2 repeats by qPCR and myocardial infarction across various subgroups. Note: Number of KIV2 repeats by qPCR were compared between the lowest quintile vs the top four quintiles. Adjusted variables included age, sex, ApoB/ApoA1 ratio. ApoB/ApoA1 ratio were treated as dichotomous variables using medians. Bonferroni-adjusted α = 0.00625 (0.05/8 tests); significance: P-interaction < 0.00625

For Lp(a) concentration (the highest quintile vs. the bottom four quintiles) and KIV-2 repeat number (the lowest quintile vs. the top four quintiles): No interactions were observed with sex, smoking status, diabetes, hypertension, daily fresh vegetables and fruits, exercise, depression, or specified life events (Figs. 3 and 4).

### Correlation between Lp(a) pathway indicators

**Fig. 5:**
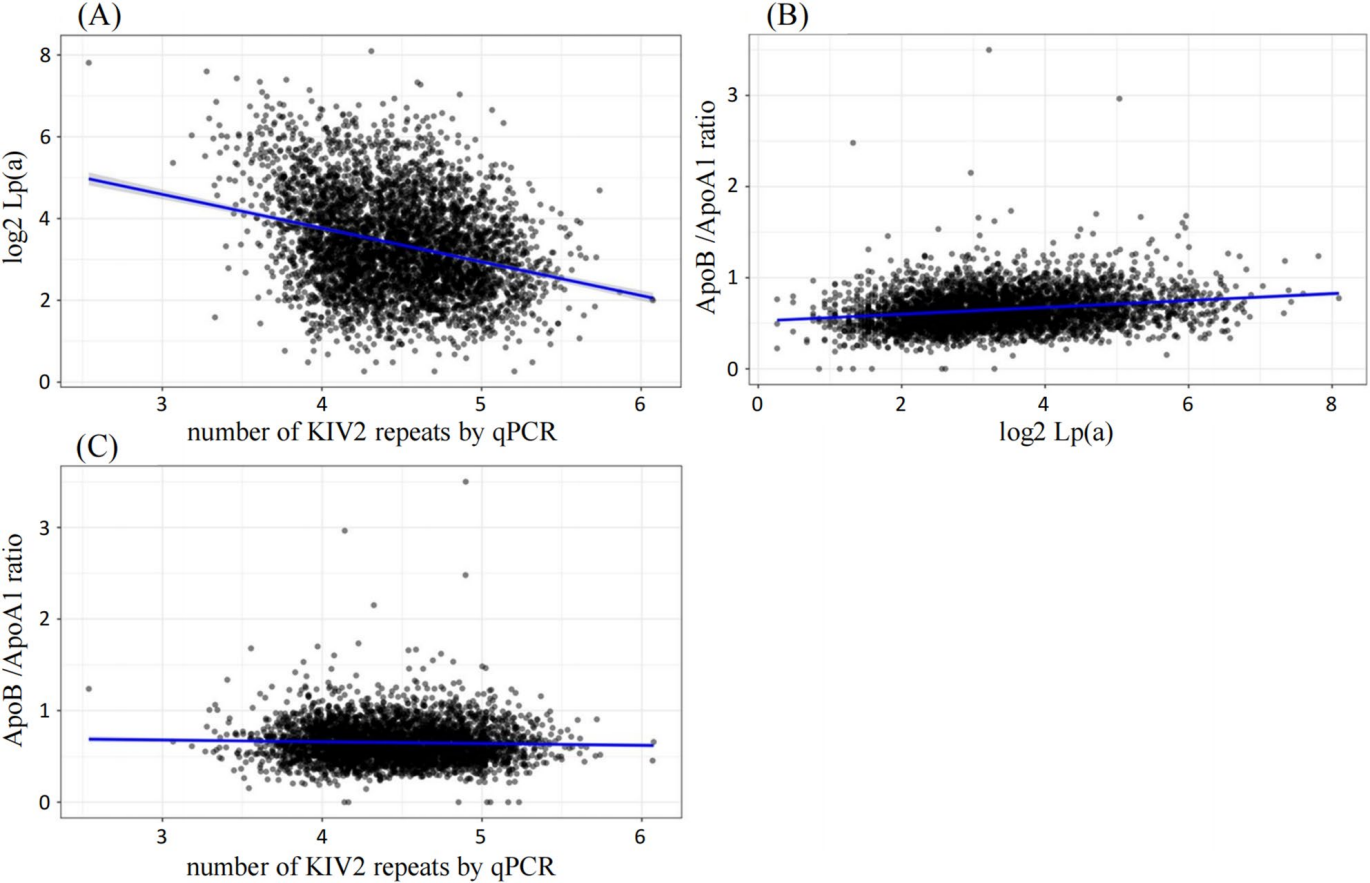
Correlation analysis between Lp(a) concentration, number of KIV2 repeats by qPCR, and ApoB/ApoA1 ratio. **A** Correlation between log2 Lp(a) and number of KIV2 repeats by qPCR. **B** Correlation between log2 Lp(a) and ApoB/ApoA1 ratio. **C** Correlation between number of KIV2 repeats by qPCR and ApoB/ApoA1 ratio

Spearman’s correlation analysis confirmed (Fig. 5): KIV-2 repeat number was negatively correlated with Lp(a) concentration (*r*=-0.323, *P* < 0.001). Lp(a) concentration was positively correlated with ApoB/ApoA1 ratio (*r* = 0.218, *P* < 0.001), while KIV-2 repeat number showed a weak negative correlation with ApoB/ApoA1 ratio (*r*=-0.043, *P* = 0.008).

### Population attributable risk (PAR)

PAR for key risk factors is shown in Table 3. Thirteen independent risk factors were included (Protective factors such as daily fresh vegetables and fruits, exercise; PAR calculated for absence of these factors). The top three were: Exercise (PAR = 28.7%), Current smoking (PAR = 27.8%), Fresh vegetables and fruit daily (PAR = 25.7%). Lp(a)-related indicators: PAR for Lp(a) (the highest quintile vs. the bottom four quintiles) was 10.3%. PAR for KIV-2 repeat number (the lowest quintile vs. the top four quintiles) was 10.4%. Combined contribution: The combined PAR for all risk factors reached 93.7% (95% CI: 53.2–99.2), indicating these risk factors account for the majority of acute myocardial infarction risk in our study population.

**Table 3:** Population attributable risk associated with the risk factors in china

Risk factor	Controls (%)	Cases (%)	PAR_1_ (95% CI) &	PAR_2_ (95% CI) #
Current smoking	27.6	42.7	20.9% (20.6 ~ 21.2)	27.8% (23.0 ~ 32.7)
Diabetes	3.2	12.1	9.3% (8.0 ~ 10.8)	9.3% (6.6 ~ 12.5)
Hypertension	22.9	38.5	20.3% (19.9 ~ 20.6)	19.8% (15.9 ~ 23.9)
Fresh vegetables and fruit daily	38.7	28.9	25.3% (25.0 ~ 25.6)	25.7% (18.9 ~ 32.2)
Exercise	14.7	10.8	27.2% (26.6 ~ 27.8)	28.7% (16.4 ~ 39.4)
Depression	8.7	17.5	9.6% (8.9 ~ 10.4)	9.7% (7.0 ~ 12.8)
Stress at work or home, Permanent	1.4	3.0	1.6% (0.1 ~ 19.6)	1.8% (0.7 ~ 3.5)
Specified life events (Two or more events)	2.9	6.0	3.2% (1.6 ~ 6.1)	3.1% (1.6 ~ 5.1)
Education (Fewer than 8 years)	45.0	54.9	18.1% (17.8 ~ 18.4)	18.7% (13.0 ~ 24.3)
ApoB/ApoA1 ratio *	20.0	37.4	21.7% (21.3 ~ 22.1)	21.7% (17.5 ~ 26.0)
LDL-C/HDL-C ratio *	20.0	32.8	16.1% (15.6 ~ 16.5)	16.6% (12.6 ~ 20.8)
Lp(a) *	20.0	28.3	10.4% (10.0 ~ 10.8)	10.3% (6.9 ~ 14.0)
number of KIV2 repeats by qPCR $	20.0	28.2	10.3% (9.9 ~ 10.8)	10.4% (7.0 ~ 14.1)
All above risk factors combined	99.2	99.9	93.7% (90.1 ~ 96.1)	93.7% (53.2 ~ 99.2)

*PAR* population attributable risk, *ApoA1*, Apolipoprotein A-1, *ApoB* Apolipoprotein B, *LDL-C* low-density lipoprotein cholesterol, *HDL-C* high-density lipoprotein cholesterol, *Lp(a) *Lipoprotein(a)

& Model unadjusted

# Model adjusted for age, sex. For protective factors (Fresh vegetables and fruit daily, Exercise), PARs are provided for the group without these factors

*PARs for ApoB/ApoA1 ratio, LDL-C/HDL-C ratio, and Lp(a) are based on a comparison of the highest quintile vs. the bottom four quintiles

$PAR for number of KIV2 repeats by qPCR is based on a comparison of the lowest quintile vs. the top four quintiles

## Discussion

Lipoprotein(a) [Lp(a)] is a unique, liver-derived atherogenic particle whose plasma concentration is predominantly determined by genetic variation at the *LPA* locus [6]. The most influential genetic determinant is the copy number variation (CNV) in the kringle IV type 2 (KIV-2) domain, where fewer repeats lead to smaller apolipoprotein(a) isoforms and, typically, higher Lp(a) levels [13]. This relationship has been robustly established through genetic epidemiology and Mendelian randomization studies, which have not only confirmed the causal role of Lp(a) in atherosclerotic cardiovascular disease (ASCVD) but also demonstrated a continuous, dose-response association between genetically elevated Lp(a) and increased risk of myocardial infarction (MI) [14]. For instance, a large Mendelian randomization study showed that a twofold increase in genetically determined Lp(a) levels was associated with a 22% higher risk of MI [15]. Despite this strong genetic link, the pathophysiological implications of the genomic KIV-2 copy number itself—beyond its role in setting Lp(a) concentration—remain incompletely understood, particularly in populations with distinct genetic backgrounds, such as the Chinese.

Based on the INTERHEART China subgroup data, this study provides novel insights into the complex relationship between Lp(a) and AMI risk in the Chinese population by being the first to employ qPCR technology in this large cohort to evaluate the independent and joint effects of genomic DNA KIV-2 repeat number and Lp(a) concentration. Our analysis demonstrates that the KIV-2 repeat copy number in genomic DNA is an independent predictor of AMI risk, an association that persisted after rigorous adjustment for Lp(a) concentration and other established risk factors. The population attributable risk for a low KIV-2 repeat number was substantial (10.4%), underscoring its potential public health significance. These results introduce a new dimension to the understanding of Lp(a) pathophysiology, suggesting that its genetic determinant may harbor risk information that is not fully captured by circulating Lp(a) concentration alone.

## Independent risk value of KIV-2 repeat number: comparison with global studies and mechanistic explanation

Our finding presents an interesting contrast with those of Paré et al. [8] and Hopewell et al. [16]. Their studies, based on immunoblotting (detecting apo(a) protein isoform size), consistently showed that after adjusting for Lp(a) concentration, the association of isoform size with MI risk was no longer significant. This strongly suggests that at the protein expression level, the impact of isoform size on cardiovascular risk is primarily mediated through regulating Lp(a) concentration.

However, this study is the first to demonstrate in a large Chinese population that the KIV-2 repeat copy number in genomic DNA, measured by qPCR, still exhibits an independent risk predictive ability (OR = 2.421) after adjusting for Lp(a) concentration. We propose that this seemingly contradictory discovery highlights the potentially different significance of KIV-2 repeats at different biological levels.

### Genomic KIV-2 copy number and Apo(a) protein isoform size: risk predictive value at different biological levels

The qPCR method used in this study captures the inherited genomic DNA blueprint of the KIV-2 repeat region and is not influenced by downstream processes such as protein expression, secretion efficiency, or degradation, thereby providing a stable reflection of an individual’s inherent genetic background. In contrast, immunoblotting detects the size of the apo(a) protein isoform actually expressed and secreted into plasma—a result subject to influence by hepatocyte secretion efficiency, proteolytic modification, and antibody affinity [9, 13]. For instance, very large isoforms (> 40 KIV-2 repeats) may escape detection due to impaired secretion, leading to misclassification of “null alleles” [17], whereas qPCR directly detects genomic DNA, avoiding such expression-related biases. Furthermore, immunoblotting has limited resolution in distinguishing small isoforms due to minor differences in electrophoretic mobility [18], while qPCR offers sensitive detection across the full spectrum of KIV-2 repeat numbers. Therefore, the KIV-2 repeat copy number measured at the DNA level may contain part of the genetic risk information that is ‘diluted’ or ‘masked’ at the protein expression level.

The observed correlation strength serves as key evidence. The relatively modest correlation we observed between genomic KIV-2 copy number and Lp(a) concentration (r = -0.323) is lower than the correlation strength reported in previous studies based on immunoblotting (e.g., r = -0.48 to -0.63 in the global INTERHEART study [8]). This indicates that in the Chinese population, the genetic information of KIV-2 copy number is not fully reflected in Lp(a) concentration. It is precisely this ‘independent’ genetic information that may directly contribute to AMI risk through mechanisms not dependent on concentration, such as by influencing protein conformation or through interactions with other vascular wall components.

### Potential for direct pathogenic mechanisms of KIV-2 repeat copy number

The independent risk associated with genomic KIV-2 copy number suggests the existence of pathogenetic mechanisms beyond its role in determining apo(a) isoform size and Lp(a) concentration. We hypothesize that the KIV-2 repeat region may influence disease susceptibility through pathways distinct from the expressed protein’s size.

One possible mechanism involves cis-regulatory effects on gene expression. The KIV-2 repeat domain may alter the local chromatin architecture and three-dimensional organization of the LPA locus, potentially modulating transcription of both the LPA gene itself and adjacent genes. For instance, KIV-2 copy number has been proposed to influence expression of the nearby PLG (plasminogen) gene, potentially creating a pro-thrombotic milieu at the transcriptional level [6, 19]. Additionally, the KIV-2 repeat region may give rise to repeat-associated non-coding RNAs that could exert regulatory functions influencing vascular inflammation or endothelial function through pathways yet to be elucidated [20, 21].

Another possibility is that the number of KIV-2 repeats may modulate the functional characteristics of Lp(a) particles by influencing the molecular structure and conformation of the synthesized apo(a) protein. The haplotype background associated with specific KIV-2 copy number may influence the structural and functional properties of the apo(a) protein. Variations in the number of kringle domains, coupled with specific single-nucleotide polymorphisms (SNPs) within the LPA haplotype, are expected to alter the protein’s architecture, potentially affecting the density, orientation, and surface exposure of functional domains. These structural nuances could, in turn, modulate the binding avidity of Lp(a) for arterial wall components, contributing to cardiovascular risk independently of Lp(a) concentration [5, 22, 23].

Consequently, our study does not negate previous findings but adds a new dimension. It suggests that for risk assessment of the Lp(a) pathway, in addition to focusing on plasma Lp(a) concentration and protein isoform size, directly detecting its root in genomic DNA – KIV-2 copy number – may provide additional, independent clinical value, especially in populations like China where Lp(a) concentrations are generally low.

## Interactive effects and clinical significance of Lp(a) concentration and KIV-2 repeat number

### Complementary value in risk prediction

This study is the first to confirm in a Chinese population that Lp(a) concentration and KIV-2 repeat number have complementary value in cardiovascular risk prediction. The correlation analysis between these two measures suggests that in addition to KIV-2 repeat number, other genetic factors (e.g., single-nucleotide polymorphisms in the LPA promoter region) or environmental factors (estrogen levels) may also contribute to the regulation of Lp(a) concentration [24, 25]. Regarding their independent risk contributions, the PAR for the highest Lp(a) quintile (>17.6 mg/dL) was 10.3%, while the PAR for the lowest KIV-2 repeat quintile (ΔCT <4.1) was 10.4%. Further spline curve analysis revealed that the combination of Lp(a) >15 mg/dL and ΔCT <4.4 (i.e., fewer KIV-2 repeats) represents a high-risk crossover zone where individual risk increases significantly. Importantly, the odds ratio (OR) for the lowest KIV-2 repeat quintile was 2.421, significantly higher than the OR of 1.784 for the highest Lp(a) quintile, underscoring the value of combined assessment of both indicators for refined risk stratification.

### Implications for current risk thresholds

The median Lp(a) concentration in the Chinese population is only 7.8 mg/dL [8], far lower than in Arab (15.3 mg/dL) and African populations (27.2 mg/dL). This study shows that even when Lp(a) concentration is within the globally defined “low-risk” range (< 30 mg/dL), its highest quintile cut-point (17.6 mg/dL) still significantly increased AMI risk (OR = 1.784). This is consistent with the risk gradient observed by Paré et al. for the Chinese population [8]. Therefore, the current international guideline-recommended Lp(a) threshold of > 50 mg/dL may not be suitable for the Chinese population. We recommend localizing the risk threshold, considering Lp(a) > 18 mg/dL (approximately the 80th percentile) as high risk for the Chinese population. Furthermore, combined screening for KIV-2 repeat number should be supplemented for individuals with Lp(a) > 15 mg/dL.

Despite the well-established causal role of Lp(a) in atherosclerotic cardiovascular disease (ASCVD) and guideline recommendations for measurement at least once in a lifetime [26, 27], the implementation of Lp(a) testing in routine clinical practice remains significantly underutilized [28]. This evidence-practice gap is particularly concerning because a substantial proportion of individuals with elevated Lp(a) may carry residual cardiovascular risk not captured by traditional lipid parameters. As highlighted in a recent review by Bosco et al., there is an urgent need for clearer clinical algorithms integrating Lp(a) measurement into residual risk assessment, particularly in the context of optimizing lipid-lowering therapy and guiding shared decision-making [29]. An ideal algorithm should consider not only Lp(a) concentration but also incorporate genetic background (e.g., KIV-2 copy number), ethnicity-specific thresholds, and interactions with other risk factors to achieve more personalized risk stratification. The results of our study directly respond to this need: we propose a combined screening strategy for the Chinese population incorporating a population-specific Lp(a) threshold and genomic KIV-2 repeat number, offering a practical step towards refined risk prediction and identification of individuals who might benefit from emerging Lp(a)-targeted therapies (e.g., antisense oligonucleotides).

## Specificity of the Chinese risk factor profile and intervention implications

### Prominent role of metabolic and non-traditional risk factors

This study reaffirms the specificity of the AMI risk factor profile in the Chinese population: the PAR for diabetes reached 9.3% (Table 3), and its risk strength (OR=3.733) exceeded that of smoking (OR=2.333) and hypertension (OR=2.009). This may be related to more significant β-cell function decline and a higher proportion of postprandial hyperglycemia in the Chinese population [30]. Psychosocial factors are severely underestimated; the PAR for depression was 9.7%, comparable to diabetes, and independent of traditional factors (adjusted OR=2.525). This aligns with the INTERHEART global analysis conclusion that "the presence of psychosocial stressors was associated with an increased risk of acute myocardial infarction, and the combined PAR for all psychological stress-related variables was 33%" [12]. Notably, the risk effects of Lp(a) and KIV-2 repeat number showed no interaction across subgroups including diabetes and depression, suggesting their risk contribution is independent of metabolic and psychological factors.

### Priority of lifestyle intervention

Although the Lp(a) pathway contributes significantly, lifestyle intervention remains the most efficient strategy. The PAR for the absence of daily vegetable and fruit intake was as high as 25.7%, and its protective effect (OR=0.596) was stronger than that of statins (typically reducing LDL-C by ~30%, equivalent to RRR ~30%). The PAR for lack of regular exercise was 28.7%, the highest among all factors. Combined with the overall joint PAR of 93.7% in this study, it suggests that maximizing the reduction of AMI burden in the Chinese population can be achieved through lifestyle interventions combined with targeted Lp(a)-lowering therapies (e.g., single-stranded antisense oligonucleotides, ASOs).

### Study Limitations

*Sample selection bias*: The case group was hospital-based, and the control group included family members of non-cardiac inpatients, potentially introducing healthy volunteer bias. KIV-2 detection limitations: qPCR cannot distinguish allele-specific repeat numbers and is insensitive to single-copy changes; future work needs to combine long-read sequencing technologies for precise genotyping. Residual confounding: Although multiple factors were adjusted for, residual confounding (e.g., undetected inflammatory markers) may affect the results. Lack of follow-up data: As a case-control study, it cannot assess the predictive value of Lp(a) and KIV-2 repeat number for long-term prognosis.

## Conclusion

This study establishes the independent and joint risk value of Lp(a) concentration and genomic KIV-2 repeat copy number in the Chinese population. Our findings reveal a risk component at the DNA level that is not fully captured by plasma Lp(a) concentration alone, thereby refining the understanding of Lp(a) pathophysiology. Considering this unique risk profile, we recommend: (1) incorporating genomic KIV-2 repeat number into high-risk screening, particularly for individuals with Lp(a) > 15 mg/dL; (2) adopting a lower, ethnicity-specific Lp(a) high-risk threshold of > 18 mg/dL for the Chinese population; (3) exploring intensive lipid-lowering strategies (e.g., PCSK9 inhibitors combined with ASOs) targeted at individuals with low KIV-2 repeat numbers.

Future research should validate the predictive value of genomic KIV-2 repeat numbers through prospective cohort studies and techniques such as long-read sequencing, while also utilizing Mendelian randomization to elucidate its causal effect independent of Lp(a) concentration. Concurrently, clinical trials of Lp(a)-lowering therapies (e.g., Pelacarsen) specifically for the Chinese population are urgently needed.

## Supplementary Information

Supplementary Material 1.

## Data Availability

Available upon request to the corresponding author.
