# Supplementary material for: Lipoprotein(a) concentration, kringle IV-2 repeat copy number, and myocardial infarction risk in Chinese populations: insights from the INTERHEART China study
Source: Lipids Health Dis. 2026 Apr 25;25:146. doi: 10.1186/s12944-026-02929-y (PMC13248369; doi:10.1186/s12944-026-02929-y)
Supplement: Supplementary file 1 — Supplementary Material 1. [file 12944_2026_2929_MOESM1_ESM.docx]

**Highlight**

1. Genomic KIV-2 repeat copy number is an independent risk factor for acute myocardial infarction in Chinese population.
2. A lower, ethnicity-specific Lp(a) risk threshold (>18 mg/dL) is proposed for the Chinese population.
3. Combined assessment of Lp(a) and KIV-2 repeats improves cardiovascular risk stratification.
4. Diabetes and depression contribute prominently to myocardial infarction risk in Chinese population.
